# The Role of HSP70 in the Protective Effects of NVP-AUY922 on Multiple Organ Dysfunction Syndrome in Endotoxemic Rats

**DOI:** 10.3389/fphar.2021.724515

**Published:** 2021-08-06

**Authors:** Pang-Yen Liu, Hsin-Hsueh Shen, Ching-Wen Kung, Shu-Ying Chen, Chia-Hsien Lu, Yen-Mei Lee

**Affiliations:** ^1^ Division of Cardiology, Department of Internal Medicine, National Defense Medical Center, Tri-Service General Hospital, Taipei, Taiwan; ^2^ Department and Graduate Institute of Pharmacology, National Defense Medical Center, Taipei, Taiwan; ^3^ Department of Nursing, Tzu Chi University of Science and Technology, Hualien, Taiwan; ^4^ Department of Nursing, Hung Kuang University, Taichung, Taiwan

**Keywords:** HSP-70, endotoxemia, MODS, septic shock, HSP-90 inhibitor

## Abstract

Sepsis is defined as a life-threatening organ dysfunction syndrome with high morbidity and mortality caused by bacterial infection. The major characteristics of sepsis are systemic inflammatory responses accompanied with elevated oxidative stress, leading to multiple organ dysfunction syndrome (MODS), and disseminated intravascular coagulation (DIC). As a molecular chaperon to repair unfolded proteins, heat shock protein 70 (HSP70) maintains cellular homeostasis and shows protective effects on inflammatory damage. HSP 90 inhibitors were reported to exert anti-inflammatory effects via activation of the heat shock factor-1 (HSF-1), leading to induction of HSP70. We evaluated the beneficial effect of HSP 90 inhibitor NVP-AUY 922 (NVP) on multiple organ dysfunction syndrome induced by lipopolysaccharide (LPS) and further explored the underlying mechanism. NVP (5 mg/kg, i.p.) was administered 20 h prior to LPS initiation (LPS 30 mg/kg, i.v. infusion for 4 h) in male Wistar rats. Results demonstrated that pretreatment with NVP significantly increased survival rate and prevented hypotension at 6 h after LPS injection. Plasma levels of ALT, CRE and LDH as well as IL-1β and TNF-α were significantly reduced by NVP at 6 h after LPS challenge. The induction of inducible NO synthase in the liver, lung and heart and NF-κB p-p65 and caspase 3 protein expression in the heart were also attenuated by NVP. In addition, NVP markedly induced HSP70 and HO-1 proteins in the liver, lung and heart after LPS injection. These results indicated that NVP possessed the anti-inflammatory and antioxidant effects on LPS-induced acute inflammation, which might be associated with HSP70 and HO-1, leading to prevent MODS in sepsis. NVP might be considered as a novel therapeutic strategy in the prevention of sepsis-induced MODS.

## Introduction

The Third International Consensus Definitions for Sepsis and Septic Shock (Sepsis-3) defines sepsis as a life-threatening multiple organ dysfunction caused by a dysfunctional body response to an infection (Singer et al., 2016). Statistically, the incidence of sepsis due to Gram-negative bacteremia in ICU patients is significantly higher than that due to other causes of infection (Abe et al., 2010). The bacterial cell walls of Gram-negative bacteria contain a compound called lipopolysaccharide (LPS), also known as endotoxin. During bacterial infection, LPS protects the bacteria from cytokines and free radicals produced by the environment and immune cells (Raetz, 1990).

Endotoxin binds to CD14 and Toll like receptor 4 (TLR4) on the surface of immune cells (monocytes and macrophages) and activates downstream cellular pathway and NF-κB signaling. The activation of NF-κB results in the secretion of proinflammatory cytokines such as tumor necrosis factor α (TNF-α), interleukin 6 (IL-6), and interleukin 1β (IL-1β) (Russell, 2006). LPS increases the expression of coagulation factors VII and VIII, the release of fibrinogen from platelets, leading to coagulopathy (Okajima, 2000). LPS triggers a rapid drop in blood pressure due to the massive production of nitric oxide (NO), leading to septic shock (Kushner and Rzewnicki, 1994). NO activates the soluble guanylyl cyclase (SGC) in smooth muscle cells to produce cyclic guanosine monophosphate (cGMP), which then activates cGMP-dependent protein kinase (PKG) and eventually leading to vasodilation (Vannini et al., 2015). Inducible nitric oxide synthase (iNOS) is abundantly expressed during inflammation. When cells are exposed to excessive NO for an extended period, nitrosative stress causes DNA damage and multiple organ failure (Martínez and Andriantsitohaina, 2009).

HSPs are a family of proteins produced by cells under stress conditions (Wang et al., 2013). In a previous study, HSP90 inhibitors were shown to improve the multiple organ dysfunction caused by sepsis (Wang et al., 2016a). HSP70, similar to HSP90, helps to refold misfolded proteins and transport unrepaired proteins to the proteasome for hydrolysis and metabolism (Jolly and Morimoto, 2000). In contrast to HSP90, which is normally present in high concentrations in cells, HSP70 is rather induced in response to stresses, such as temperature, salinity, or hypoxia (Feder and Hofmann, 1999).

In sepsis, endoplasmic reticulum stress and the presence of damaged mitochondria lead to release of cytochrome c into cytosol and increase of oxidative stress. The binding of LPS and TLR4 enhances autophagy by conversion of LC3-I to LC3-II to initiate formation and lengthening of the autophagosome. Autophagy prevents further deterioration of the pro-inflammatory response and minimalized oxidative damage by sequestering malfunctioning organelles.

NVP-AUY922, also known as Luminespib, is a structural derivative of the fungal antibiotic radicicol (RD). Five thousand structural derivatives of RD were screened, and CCT018159 was selected as the most effective inhibitor of the ATP hydrolase in HSP90, thereby inactivating HSP90. The drug was developed from structural modification of CCT01859 as a new generation of HSP90 inhibitors (Cheung et al., 2005; Eccles et al., 2008). In an inflammation-related study on the mouse macrophage cell line RAW264.7, researchers found that RD could inhibit the NF-κB and MAPK pathways activated by LPS, inhibit iNOS induction, reduce NO production, and reduce the production of pro-inflammatory cytokines such as TNF-α and IL-1β (Jeon et al., 2000). In a cancer cell experiment, NVP-AUY922 was found to inhibit the NF-κB pathway to reduce the inflammatory response of cancer cells, indicating the anti-inflammatory potential of the drug (Walsby et al., 2013).

The aim of this study was to observe the effect of NVP-AUY922, a next-generation HSP90 inhibitor, on the prevention of endotoxin-induced multiple organ failure in an animal model of endotoxemia, and to investigate whether it could inhibit the activation of the NF-κB signaling pathway, inhibit the release of pro-inflammatory cytokines, generate antioxidants, reduce apoptosis, activate autophagy, and reduce disseminated intravascular coagulation (DIC) to improve sepsis.

## Materials and Methods

### Animals and LPS Animal Model

*In vivo* experiments were performed on 8-week-old male Wistar rats weighing 275–300 g purchased from BioLASCO Taiwan Co., Ltd. To maintain environmental quality, all animals were housed in the Laboratory Animal Center of National Defense Medical Center at a temperature of 22 ± 1°C under a 12-h light–dark cycle. This experiment was reviewed and approved by the Animal Experiment Management Team of the National Defense Medical Center (Certificate No.: IACUC-18-230). The rats were anesthetized with pentobarbital (25 mg/kg) and urethane (0.6 g/kg) via intraperitoneal injection, and after the animals were anesthetized, a median excision of ∼2 cm was performed on their necks. A polyethylene tube (PE240) was used for tracheal intubation to facilitate sputum extraction and to maintain respiratory function. A polyethylene tube (PE50) filled with 0.9% saline was used to intubate the left femoral vein, and a polyethylene tube (PE50) filled with 10 IU/ml Heparin was used to intubate the left femoral artery to record arterial blood pressure. The arterial cannula was connected to a pressure transducer using a three-way valve, and the signal was connected to a physiology multi-recorder (PowerLab, Polygraph Instrument, Australia) to record blood pressure, mean arterial pressure (MAP), and heart rate (HR). A temperature sensor was inserted into the rectum to record the core temperature (Tco). LPS 30 mg/kg (dissolved in 9 ml of saline) was administered via the femoral vein for 4 h (2.25 ml/h). Blood (0.5 ml each time) was collected from the femoral artery at 0, 2, 4, and 6 h with equal volume of normal saline replenished. Blood glucose, biochemical values, and cytokine changes in plasma were measured. If the blood pressure of the rats dropped to 30 mmHg during the experiment, the hypotension was irreversible, the rats were defined as dead.

### Experimental Design

The animals were divided into four groups: (Singer et al., 2016) control group: 0.9% saline was administrated as a control (Abe et al., 2010). NVP-AUY922 administration group (NVP group): NVP-AUY922 (5 mg/kg) and saline (1 ml) was administered via intraperitoneal injection 20 h before experiment (Raetz, 1990). Endotoxin group (LPS group): LPS (30 mg/kg; dissolved in 9 ml of saline) was administered via the femoral vein for 4 h (2.25 ml/h) to establish a model of severe endotoxemia (Russell, 2006). Pre-administration of NVP-AUY922 + endotoxin LPS group (NVP + LPS group): NVP-AUY922 (5 mg/kg) was administered via intraperitoneal injection 20 h before. LPS (30 mg/kg; dissolved in 9 ml of saline) was administered via the femoral vein for 4 h (2.25 ml/h). The rats were sacrificed for organ retrieval 6 h after the experiment in each group.

### Measurement of Blood Glucose, Biochemical Values of Organ Function Indicators, and Coagulation Function

Blood (10 μl) was collected from the femoral artery at 0 (baseline) 2, 4, and 6 h after LPS infusion. Blood glucose level was measured by using Bayer’s Ascensia Elite Blood Glucose Monitoring System (Bayer HealthCare LTD., Leverkusen, Germany). Alanine aminotransferase (ALT) for liver function, creatinine (CRE) for kidney function, creatine phosphokinase (CPK) for skeletal muscle impairment, and lactate dehydrogenase (LDH) for cell damage were determined. Approximately 0.3 ml of whole blood was collected and stored in a moist centrifuge tube containing 4% sodium citrate (whole blood: sodium citrate = 9:1 to prevent blood clotting), slowly warmed, and mixed well with sodium citrate, using an automated hematology analyzer (Sysmex KX-21; TOA Medical Electronics Co., Kobe, Japan) to measure platelet counts. The difference of platelet count 6 h after LPS administration from baseline was calculated.

### Determination of Inflammation-Related Cytokines

Antibodies (TNF-α, IL-1β, IL-6, and MIP-2) were selected for the assay, and all standards and samples were tested using the duplex method. After preparation of standard and sample, the sample was diluted with Calibrator Diluent and mixed well. Wash buffer was rinsed five times and the wash solution of each well was blotted dry. The beads were mixed and placed in a 96-well plate and left to stand for 10 min. Next, 50 μl of the sample was added to the plate to react with the beads for 40 min, followed by the addition of the biotin labeled antibody to react for 40 min. The signal was amplified by connecting the PE-labeled streptavidin to the biotin label, and two fluorescence signals were generated at the end of the reaction. Intensity of the fluorescence signal was detect by using the Bio-plex^®^ MAGPIX™ multiplex reader (Bio-Rad Laboratories, Inc.) and the liquid flow system. The final green laser signal was rapidly processed into a digital signal and analyzed to determine the level of each protein.

### Expression of Inflammation-Related Proteins in Severe Endotoxemia

Western blot was used for measurement of protein expressions 6 hours after LPS-induced severe endotoxemia. We assessed the expression of (Singer et al., 2016) the inflammation-related protein iNOS in the rat heart, lung, and liver, and the NF-κB pathway activation index proteins phosphorylated p65 (p-p65) and phosphorylated IκB (*p*-IκB) in cardiac myocytes; (Abe et al., 2010) the inducible protective proteins Hsp70, HSF-1, and heme oxygenase (HO-1); (Raetz, 1990) the apoptosis marker caspase 3; (Russell, 2006) the determination of cellular autophagy marker LC3-II; (Okajima, 2000) the internal control proteins α-actin, β-actin, and Histone 1. The Samples were homogenized and then shaken for 1 hour in a cold room at 4°C. Samples were then centrifuged at 10,000 g and 4°C for 30 min, and the supernatant was collected for quantification. 20 μg of each sample was taken and diluted in SDS and protein loading dye in a 4:1 ratio and heated at 95°C for 5 min to denature the protein. Samples were fractionated by 10% SDS polyacrylamide gel electrophoresis and transferred to nitrocellulose membrane. The membranes were then blocked in TBST containing 5% BSA and incubated in primary antibodies against iNOS (610432, 1:1,000 dilution, BD), p-p65 (3033, 1:1,000 dilution, CST), *p*-IκB (2859, 1:1,000 dilution, CST), Hsp70 (C92F3A-5, 1:1,000 dilution, Enzo Life Sciences), HSF-1 (4356, 1:1,000 dilution, CST), HO-1 (sc-1089, 1:1,000 dilution, Santa Cruz Biotechnology), caspase 3 (31A1067, 1:1,000 dilution, Enzo Life Sciences), and LC3 (GTX48643, 1:1,000 dilution, GeneTex) overnight at 4°C. Subsequently, membranes were incubated in anti-mouse IgG (7076, 1:1,000 dilution, CST) or Anti-rabbit IgG (7074, 1:1,000 dilution, CST) conjugated to horseradish peroxidase overnight at 4°C or for 1 h at room temperature and then scanned using the UVP Plus luminescence camera system.

### Hematoxylin and Eosin Staining

Collected lung and liver were fixed in 10% paraformaldehyde overnight and processed for paraffin histology with 4-μm sections stained with hematoxylin and eosin staining was used. In brief, the sections were stained in hematoxylin for 2 min, and then washed with water for 15 min. Then the sections were stained in eosin for 60 s, dehydrated, mounted and then reviewed using light microscopy.

### Statistical Analysis

All experimental data were expressed as mean ± standard error (SEM). The numerical comparisons among the groups were performed using one-way ANOVA and the Student-Newman–Keuls test was used for post-hoc comparison. Survival analysis was performed using the log-rank test.

## Results

### Effects of NVP on Survival Rate and Physiological Parameters of Endotoxemic Rats

**FIGURE 1:**
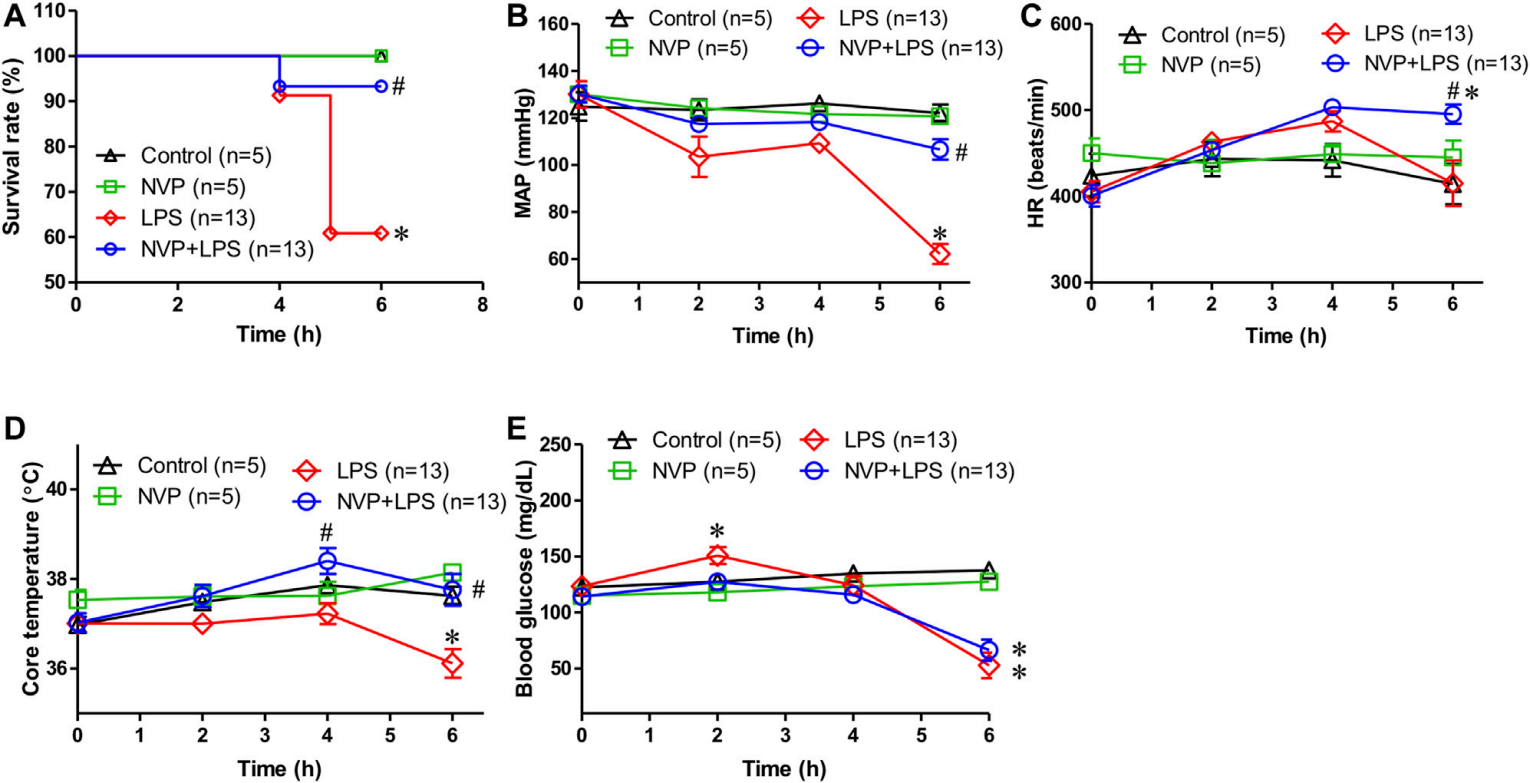
Effects of NVP-AUY922 on physiological parameters. **(A)** Survival rate. Effects of NVP-AUY922 pre-treatment on survival rate during endotoxemia. **(B)** Mean arterial pressure. Effects of NVP-AUY922 pre-treatment on mean arterial pressure during endotoxmia. MAP: mean arterial blood pressure. **(C)** Heart rate. Effects of NVP-AUY922 pre-treatment on heart rate during endotoxmia. HR: heart rate. **(D)** Body temperature. Effects of NVP-AUY922 pre-treatment on core temperature during endotoxmia. **(E)** Blood glucose. Effects of NVP-AUY922 pre-treatment on blood glucose during endotoxmia. NVP: NVP-AUY922 5 mg/kg. Values are expressed as mean ± SEM. *p < 0.05 vs. Control group, #*p* < 0.05 vs. LPS group.

In Figure 1A, the survival rate of control and NVP groups were 100% during the experimental period, (5/5). The survival rate in the LPS group was 60.8% and was significantly lower than that in the control and NVP groups (*p* < 0.05) (13/21). In the NVP + LPS group, one rat died at the sixth hour after the start of LPS infusion (survival rate = 13/14, 93.3%). Figure 1B shows that each group was observed for 6 h after the injection of saline and drugs, and the MAP was recorded at each time point during the experiment. There was no significant difference in the baseline average arterial pressure among the five groups. In the control and NVP groups, there was no significant change in MAP during the 6-h observation period. In the LPS group, the blood pressure decreased slightly after the start of LPS infusion, and by 6 h, the blood pressure dropped to 62.2 ± 15.0 mmHg, significantly lower than 122.1 ± 8.0 mmHg observed in the control group (*p* < 0.05). The blood pressure of the NVP + LPS group was 106.6 ± 15.7 mmHg at 6 h after the start of LPS infusion, which was significantly higher than that in the LPS group (*p* < 0.05). As shown in Figure 1C, there was no significant difference in the baseline HR among the four groups; there was no significant change in HR in the control and NVP groups during the 6-h observation period. In the LPS group, the HR started to increase slightly 2 h after the start of LPS infusion, subsequently starting to decrease after 4 h, with a HR of (415.2 ± 95.2 beats/min) at 6 h; there was no significant difference from the control group. In the NVP + LPS group, 2 h after the start of LPS infusion, the HR started to increase slightly; by 4 h the HR (503.3 ± 26.9 beats/min) was the highest among the four groups; and by 6 h the HR (495.4 ± 40.4 beats/min) was significantly higher than that in the LPS group (*p* < 0.05). Figure 1D indicates no significant difference in the baseline rectal temperature among the four groups. In the control and NVP groups, there was no significant change in the rectal temperature during the 6-h observation period; in the LPS group, the body temperature decreased slightly after the start of LPS infusion, and the rectal temperature decreased significantly at 6 h (36.1 ± 1.1°C at the sixth hour), which was significantly lower than that in the control group (37.6 ± 0.5°C) (*p* < 0.05). In the NVP + LPS group, 2 h after the start of LPS infusion, the rectal temperature began to rise slightly to 38.4 ± 1.0°C at 4 h, significantly higher than that in the LPS group (*p* < 0.05), and then dropped to the same level as that in the control group at the sixth hour (37.8 ± 1.3°C), significantly higher than that in the LPS group (*p* < 0.05). Thus, the pre-administration of NVP could avoid the thermoregulation disorder caused by LPS. As shown in Figure 1E, there was no significant difference in the baseline blood glucose levels among the four groups. The control and NVP groups showed no significant changes in blood glucose during the 6-h observation period. However, 2 hours after LPS administration, the blood glucose values in the LPS group were significantly higher than those in the control group (*p* < 0.05), with no significant differences among the NVP, NVP + LPS, and control groups (control: 127.4 ± 13.9 mg/dL; NVP: 118.0 ± 11.6 mg/dL; LPS: 150.9 ± 27.7 mg/dL) (*p* < 0.05). The degree of elevated blood glucose is one of the initial indicators of induced sepsis. After the fourth hour, the blood glucose in the LPS and LPS + NVP groups started to decrease and was significantly lower than that in the control group by the sixth hour (control: 137.6 ± 15.1 mg/dL; NVP: 127.4 ± 5.0 mg/dL; LPS: 52.9 ± 41.3 mg/dL; NVP+LPS (*p* < 0.05), although there was no significant difference between the LPS and NVP + LPS groups. This indicates that NVP pretreatment did not improve LPS-induced blood glucose loss.

### Effects of NVP on LPS-Induced Organ Toxicity and Cytotoxicity

**FIGURE 2:**
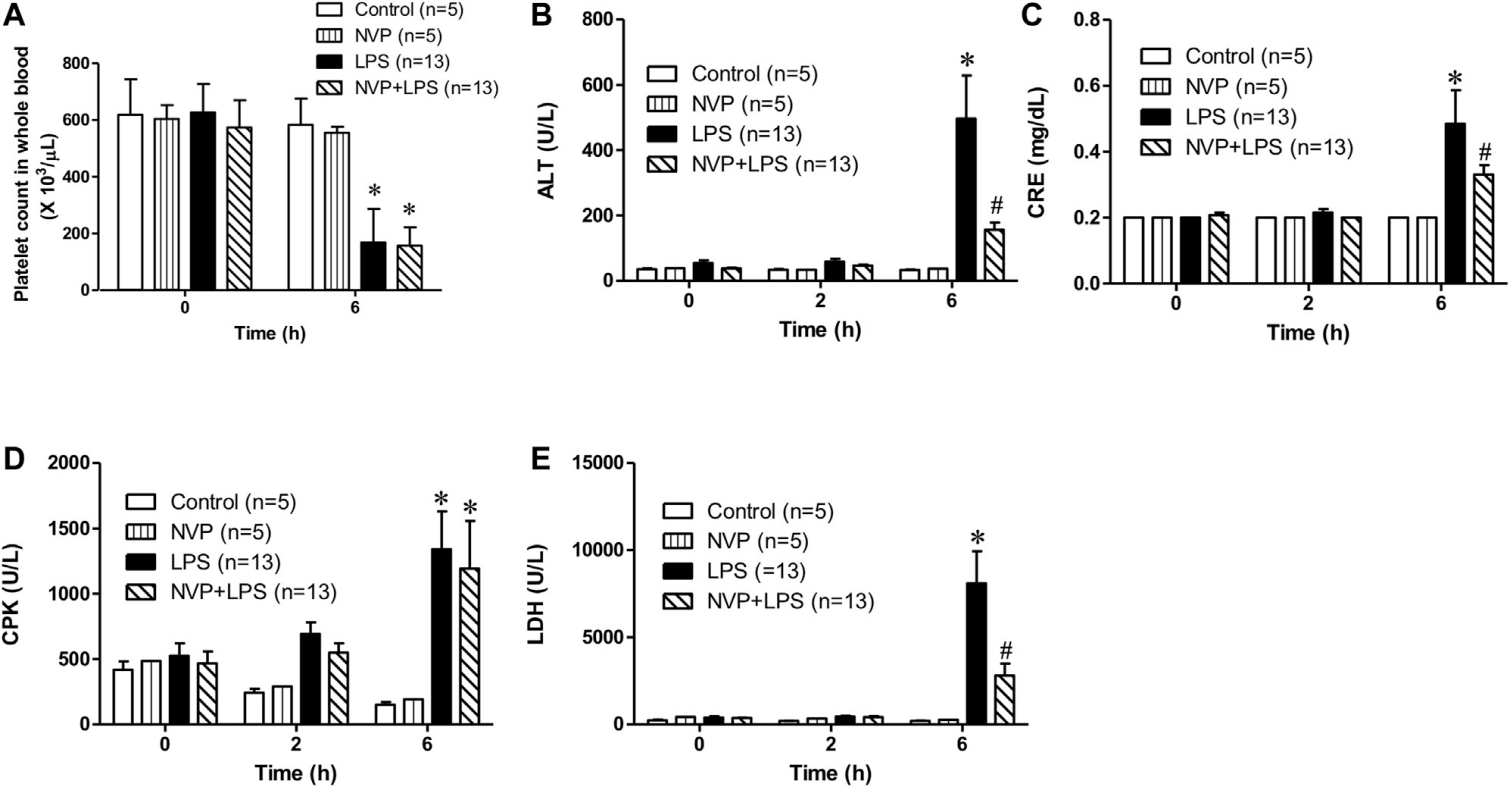
Effects of NVP-AUY922 on organ damage markers. **(A)** Platelet count. Effects of NVP-AUY922 pre-treatment on platelet count in whole blood 6 h after rats being subjected to LPS administration. **(B)** Liver function test. Effects of NVP-AUY922 on plasma levels of alanine aminotransferase. ALT: alanine aminotransferase. **(C)** Renal function test. Effects of NVP-AUY922 on plasma levels of creatinine. CRE: creatinine. **(D)** Skeletal muscle injury. Effects of NVP-AUY922 on plasma levels of creatine phosphokinase. CPK: creatine phosphokinase. **(E)** Cytotoxicity. Effects of NVP-AUY922 on plasma levels of lactate dehydrogenase. LDH: lactate dehydrogenase. NVP: NVP-AUY922 5 mg/kg. Values are expressed as mean ± SEM. *p < 0.05 vs. Control group, #*p* < 0.05 vs. LPS group.

Figure 2A suggests no significant difference in the baseline platelet count among the groups. The platelet count in the LPS group was significantly lower than that in the control group 6 h after the initiation of LPS infusion. In the NVP (NVP + LPS) pretreatment group, the platelet count was also reduced 6 h after the start of LPS infusion, and there was no significant difference compared to LPS, demonstrating that NVP did not reduce LPS-induced platelet loss. As shown in Figure 2B, there was no significant difference in the baseline ALT, a liver function indicator, among the four groups. Figure 2B shows that the plasma ALT values increased with the time of LPS administration, and at 6 h after LPS administration, the plasma ALT value in the LPS group was 496.4 ± 47.5 U/L, which was significantly higher than that in the control group (32.8 ± 4.4 U/L) (*p* < 0.05). The ALT value in the NVP + LPS group at 6 h after LPS infusion was 156.3 ± 79.0 U/L, significantly lower than that in the LPS group (*p* < 0.05), indicating that NVP could improve the liver injury caused by LPS. Figure 2C reveals no significant difference in the baseline CRE among the groups. Six hours after the start of LPS infusion, the plasma CRE value in the LPS group was 0.5 ± 0.4 mg/dL, significantly higher than that in the Control group (0.2 ± 0.0 mg/dL) (*p* < 0.05). In the NVP + LPS group, the plasma CRE value was 0.3 ± 0.1 mg/dL 6 h after the start of LPS infusion, significantly lower than that in the LPS group (*p* < 0.05). The results in Figure 2D shows that 6 h after the start of LPS infusion, the plasma CPK value in the LPS group was 1,341.2 ± 305.5 U/L, which was significantly higher than that in the control group (150.0 ± 48.0 U/L) (*p* < 0.05). In the NVP + LPS group, the plasma CPK value was 1,309.505 ± 116.7 U/L 6 h after the start of LPS infusion, significantly higher than that in the control group (*p* < 0.05), but there was no significant difference compared with the LPS group. Figure 2E exhibits that 6 h after the start of LPS infusion, the plasma LDH value in the LPS group (8,102.3 ± 774.2 U/L) was significantly higher than that in the control group (189.8 ± 74.7 U/L) (*p* < 0.05). The plasma LDH value (2,802.8 ± 335.7 U/L) in the NVP + LPS group was significantly lower than that in the LPS group 6 h after the start of LPS infusion (*p* < 0.05).

### Effects of NVP on LPS-Induced Release of Various Cytokines

**FIGURE 3:**
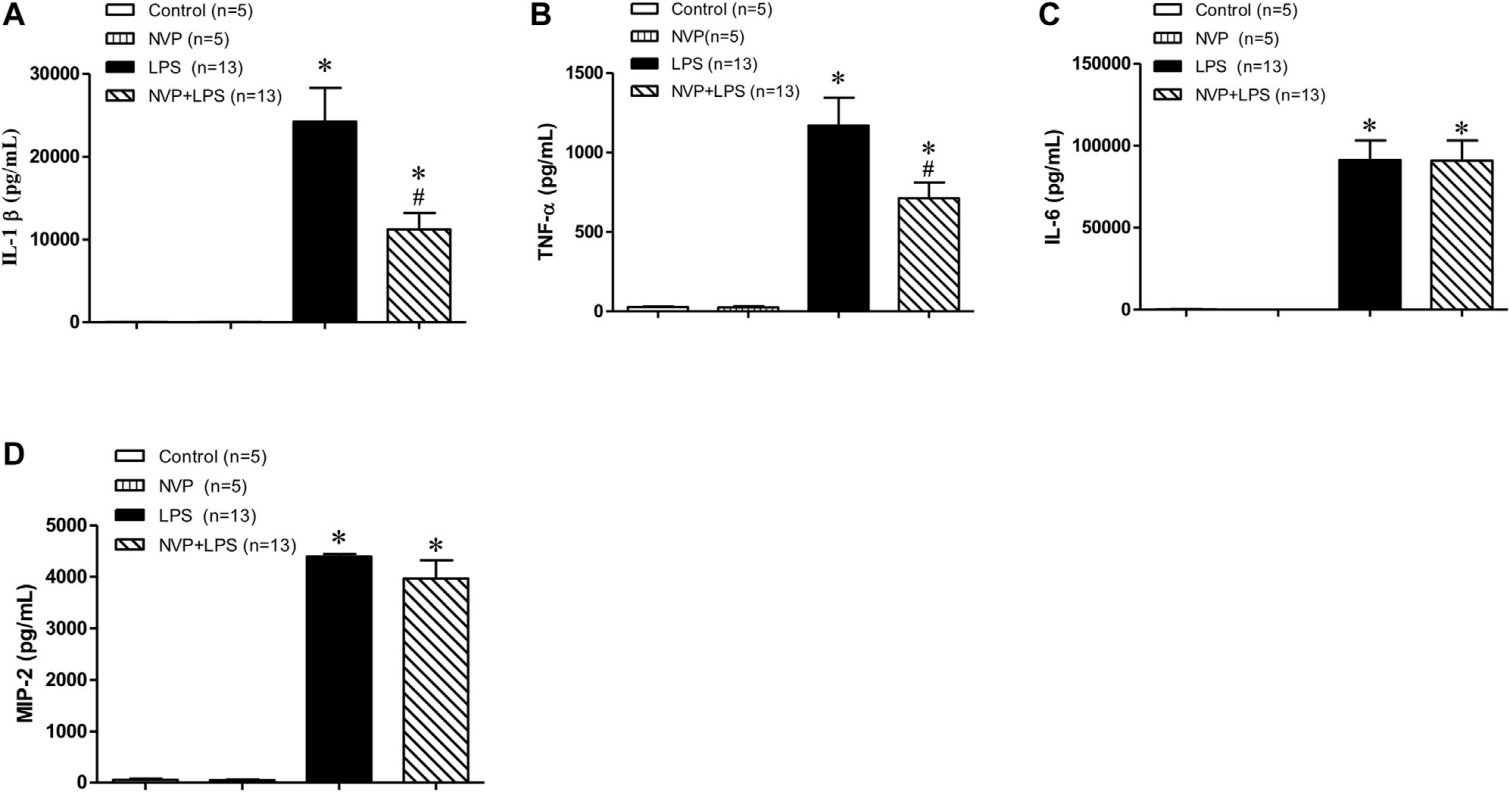
Effects of NVP-AUY922 on pro-inflammatory cytokines. **(A)** Plasma cytokine IL-1β. Effects of NVP-AUY922 on plasma levels of pro-inflammatory cytokine interleukin-1β (IL-1β) 6 h after LPS administration in rats. **(B)** Plasma cytokine TNF-α. Effects of NVP-AUY922 on plasma levels of tumor necrosis factor α (TNF-α) 6 h after LPS administration in rats. **(C)** Plasma IL-6. Effects of NVP-AUY922 on plasma levels of pro-inflammatory cytokine interleukin-6 (IL-6) 6 h after LPS administration in rats. **(D)** Plasma MIP2. Effects of NVP-AUY922 on plasma levels of pro-inflammatory cytokine macrophage inflammatory protein (MIP-2) 6 h after LPS administration in rats. NVP: NVP-AUY922 5 mg/kg. Values are expressed as mean ± SEM. *p < 0.05 vs. Control group, #*p* < 0.05 vs. LPS group.

As shown in Figures 3A,B-, there was no significant difference in the plasma levels of IL-1β and TNF-α between the control and NVP groups 6 h after the experiment; however, 6 h after LPS infusion, the plasma levels of IL-1β and TNF-α increased significantly in the LPS group, which was statistically different from those in the control group (*p* < 0.05); NVP + LPS group significantly decreased the plasma levels of IL-1β and TNF-α 6 h after LPS administration, as compared to the LPS group (*p* < 0.05). In addition, the plasma concentrations of IL-6 and MIP-2 in the LPS group significantly increased and were statistically different from those in the control group (*p* < 0.05). The plasma concentrations of IL-6 and MIP-2 in the NVP + LPS group were not significantly different from those in the LPS group 6 h after the start of LPS infusion (Figures 3C,D).

### Effects of NVP on the Protein Expression of LPS-Induced Inflammatory Pathways

**FIGURE 4:**
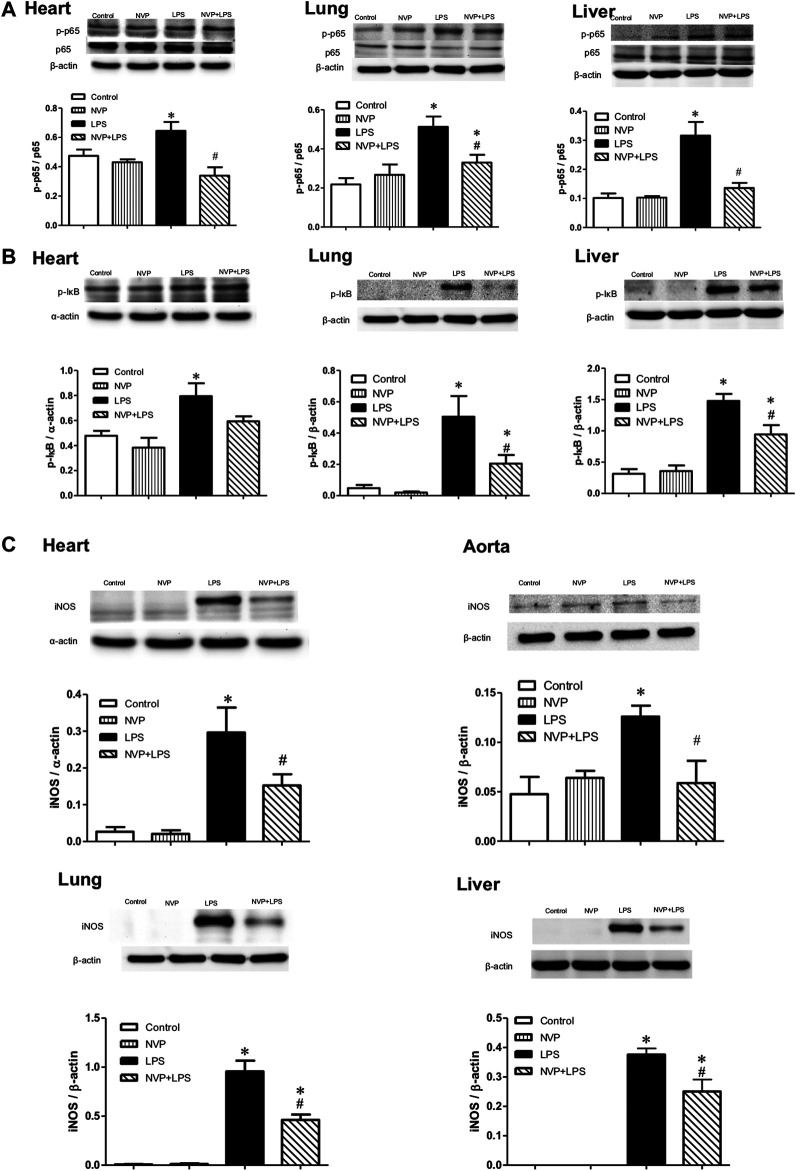
Effects of NVP-AUY922 on proinflammatory proteins. **(A)** Phosphorylated NF-κB p65 protein expressions in heart, lung and liver. Effects of NVP-AUY922 pre-treatment on phosphorylated NF-κB p65 (p-p65) in hearts, lungs, and livers 6 h after LPS administration in rats. **(B)** Phosphorylated IκB protein expressions in heart, lung and liver. Effects of NVP-AUY922 pre-treatment on phosphorylated IκB (*p*-IκB) in hearts, lungs, and livers 6 h after LPS administration in rats. **(C)** Protein expression of iNOS in cardiovascular system, lung and liver. Effects of NVP-AUY922 pre-treatment on iNOS in hearts, aortas, lungs and livers 6 h after LPS administration in rats. NVP: NVP-AUY922 5 mg/kg. Values are expressed as mean ± SEM. *p < 0.05 vs. Control group, #*p* < 0.05 vs. LPS group.

Figures 4A,B- show the expression levels of the p-p65 protein in the heart (A), lung (B), and liver (C) 6 h after LPS administration. There were no differences between the Control and NVP groups in the three organs. A significant increase in the expression levels of the p-p65 protein was observed in the LPS group compared to the Control group (*p* < 0.05). Six hours after the start of LPS infusion in NVP the (NVP + LPS group) pretreatment, the expression levels of the p-p65 protein in the tissues were significantly lower than those in the LPS group (*p* < 0.05). The expression levels of *p*-IκB protein in the LPS group were significantly increased compared to those in the Control group (*p* < 0.05). Six hours after the start of LPS infusion in NVP the (NVP + LPS group) pretreatment, the expression levels of *p*-IκB protein in the heart was were lower than that in the LPS group, but the was not statistically significant. The expression levels of *p*-IκB protein in the lung and liver were significantly lower than those in the LPS group (*p* < 0.05). Figure 4C demonstrates the expression levels of iNOS protein in the heart, aorta, lung, and liver cells. There was no intergroup difference in the four tissues between the Control and NVP groups. iNOS expression in the heart, lung, and liver was significantly higher in the LPS group than in the Control group 6 h after the start of LPS infusion (*p* < 0.05). NVP-AUY922 NVP p (NVP + LPS group) pretreatment showed resulted in a significant decrease in iNOS expression in the heart, lung, and liver 6 h after the start of LPS infusion, which was significantly lower than that in the LPS group (*p* < 0.05).

### Effects of NVP on the Expression of Anti-Inflammatory Proteins

Figures 4A,B show the expression levels of the p-p65 protein in the heart (A), lung (B), and liver (C) 6 h after LPS administration. There were no intergroup differences between the control and NVP groups in the three organs. A significant increase in the expression levels of the p-p65 protein was observed in the LPS group compared to the control group (*p* < 0.05). Six hours after the start of LPS infusion in the NVP + LPS group, the expression levels of the p-p65 protein in the tissues were significantly lower than those in the LPS group (*p* < 0.05). The expression levels of *p*-IκB protein in the LPS group were significantly increased compared to those in the control group (*p* < 0.05). Six hours after the start of LPS infusion in the NVP + LPS group, the expression levels of *p*-IκB protein in the heart were lower than that in the LPS group, but a statistical difference was not achieved. The expression levels of *p*-IκB protein in the lung and liver were significantly lower than those in the LPS group (*p* < 0.05). Figure 4C demonstrates the expression levels of iNOS in the heart, aorta, lung, and liver cells. There was no intergroup difference in the four tissues between the control and NVP groups. iNOS expression in the heart, lung, and liver was significantly higher in the LPS group than in the control group 6 h after the start of LPS infusion (*p* < 0.05). NVP-AUY922 pretreatment resulted in a significant decrease in iNOS expression in the heart, lung, and liver 6 h after the start of LPS infusion, which was significantly lower than that in the LPS group (*p* < 0.05).

### Effects of NVP on the Expression of Autophagy Indicator LC3-II

**FIGURE 5:**
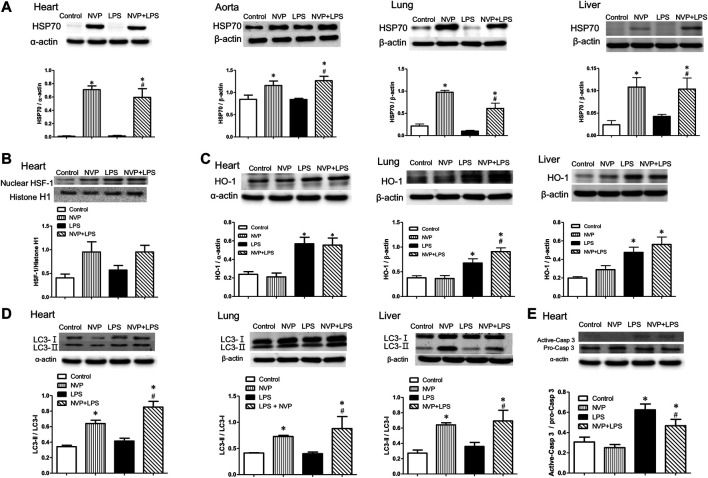
Effects of NVP-AUY922 on anti-inflammatory protein expressions. **(A)** Effects of NVP-AUY922 pre-treatment on HSP70 in hearts, aortas, lungs and livers 6 h after LPS administration in rats. **(B)** Effects of NVP-AUY922 pre-treatment on nuclear HSF-1 expression in hearts 6 h after LPS administration in rats. **(C)** LPS effects on autophagy in multiple organs. Effects of NVP-AUY922 pre-treatment on HO-1 in hearts, lungs, and livers 6 h after LPS administration in rats. **(D)** LPS effects on apoptosis in multiple organs. Effects of NVP-AUY922 pre-treatment on LC3 protein expression in hearts, lungs, and livers 6 h after LPS administration in rats. **(E)** Effects of NVP-AUY922 pre-treatment on the ratio of active form caspase 3 and pro-form caspase 3 protein expression of hearts 6 h after LPS administration in rats. NVP: NVP-AUY922 5 mg/kg. HSF-1: heat shock factor-1. HO-1: heme oxygenase-1. Values are expressed as mean ± SEM. *p < 0.05 vs. Control group, #*p* < 0.05 vs. LPS group.

Figure 5D shows the expression of LC3-II protein in heart, lung, and liver cells. There were no intergroup differences between the control and NVP groups in the three organs. Six hours after the start of LPS infusion, the expression of LC3-II in the heart, lung, and liver was slightly higher in the LPS group than in the control group. The NVP + LPS group showed significantly higher expression of LC3-II in the three organs compared to the LPS group (*p* < 0.05). Thus, it can be concluded that NVP-AUY922 pretreatment can increase autophagy during endotoxemia.

### Effects of NVP on the Expression of Caspase 3, a Marker of Cardiac Apoptosis

Figure 5E presents the protein expression levels of inactive Caspase 3 (Pro-Caspase-3) and activated Caspase 3 (Activated-Caspase-3) in cardiomyocytes, showing that there was no significant difference in caspase 3 protein expression in cardiomyocytes between the control and NVP groups. The expression of activated caspase-3 protein in the LPS group was significantly higher than that in the control group (*p* < 0.05) 6 h after the start of LPS infusion. In the NVP + LPS group, the expression of activated caspase-3 protein in cardiomyocytes was reduced 6 h after the start of LPS infusion, and was significantly lower than that in the LPS group (*p* < 0.05). The results of this experiment showed that NVP-AUY922 could improve LPS-induced apoptosis of cardiomyocytes.

### The Impact of NVP in LPS Induced Lung and Liver Injury

**FIGURE 6:**
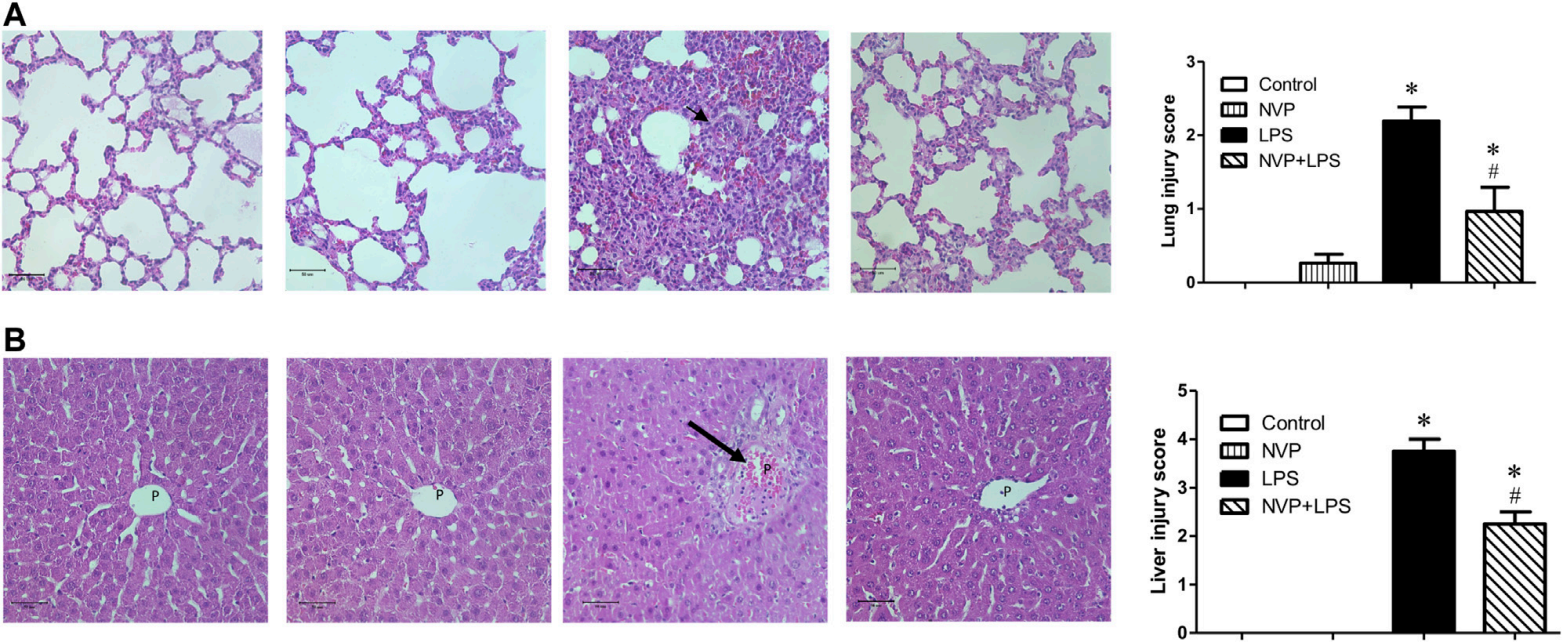
Effects of NVP-AUY922 pretreatment on histology of lung(A) and liver(B) sections. Sections were stained with hematoxylin and eosin, each ×400 (original magnification). Panels from left to right were control, NVP, LPS and NVP+LPS group, respectively. Short arrow in **(A)** indicates infiltration of the inflammatory cells. Long arrow in **(B)** indicates degeneration and necrosis of the hepatocytes. P, portal vein space. Scale bar: 50 μm. Values are expressed as mean ± SEM. *p < 0.05 vs. Control group, #*p* < 0.05 vs. LPS group.

In lung tissue, LPS group showed significant neutrophil infiltration, thickened alveolar wall and interstitial edema (short arrow in Figure 6A). NVP+LPS group demonstrated improvement in neutrophil infiltration and edema. In liver, LPS group showed degeneration and necrosis of the hepatocytes (long arrow in Figure 6B). With NVP, the hepatocyte injuries by LPS were attenuated (Figure 6B).

## Discussion

By pre-administration of NVP-AUY922, an HSP90 inhibitor, the LPS-induced inflammatory response induced and multiple organ failure were ameliorated in a rat model of severe endotoxemia. NVP-AUY922 pretreatment inhibited the inflammatory NF-κB pathway and reduced the expression of iNOS and inflammation-related cytokines, which resulted in the alleviation of inflammation, improvements in abnormal liver and kidney function, reduction of cytotoxicity, and reduction of apoptosis in lung and cardiac tissue. The present study offers new insights into the beneficial effects of the NVP-AUY922 were dependent on induction of HSP70 and HO-1 in the heart, lung and liver, and preservation of cellular autophagy by enhancing LC3-II to LC3-I ratio.

The mortality rate of sepsis is as high as 12–15% in clinical settings (Fleischmann et al., 2016; Fleischmann-Struzek et al., 2018). In the present results, the survival rate of rats in the NVP+LPS group was significantly higher than that in the LPS group (Figure 1A), indicating that NVP-AUY922 is protective in endotoxemia (Figure 1A). Shock often occurs at the end of sepsis and increases the mortality rate as a result of insufficient tissue perfusion (MacLean et al., 1967). In the present study, blood pressure in the LPS group decreased significantly, while the NVP + LPS group showed improvements in hypotension (Figure 1B). In endotoxemia, NVP-AUY922 increases HR and may enhance sympathetic activity, which helps to maintain blood pressure and increases HR. Previous studies have reported that the effect of LPS on blood pressure is divided into two periods; initially, LPS stimulates the production of substances that mediate vascular tension (e.g., bradykinin, platelet activating factor, and endothelin), causing the endothelial nitric oxide synthase (eNOS) in vascular endothelial cells to produce trace amounts of NO, resulting in vasodilation and a temporary decrease in blood pressure (Fairchild and O'Shea, 2010; Kakihana et al., 2016). This change in blood pressure activates the pressure-sensing reflex, which increases the HR to maintain blood pressure. As LPS stimulates the activation of macrophages and neutrophils, large amounts of cytokines, including TNF-α and IL-6, are released, which induces iNOS and NO production to cause endothelial smooth muscle relaxing, leading to septic shock. Reactive oxygen species (ROS) are also produced during sepsis, or combined with excess NO to form peroxynitrite, which damages vascular smooth muscle cells. Cardiomyocyte damage and apoptosis due to ROS and cytokines lead to low cardiac output and HR (Fairchild and O'Shea, 2010; Vannini et al., 2015).

Physiologic patterns of temperature change could be an early indicator of sepsis (Drewry et al., 2013). The present experiment showed that the body temperature decreased significantly at the fourth hour after the start of LPS infusion, while the NVP + LPS group showed a slight increase. Six hours after LPS infusion, the body temperature of the LPS group was significantly lower than that of the control group, and the NVP + LPS group was comparable to that of the control group. Thus, NVP-AUY922 pretreatment could prevent dysregulation of thermal control induced by LPS (Figure 1D).

In sepsis, the inflammatory response and coagulation are mutually influential (Allen et al., 2015). Previous studies reported formation of microvascular blood clots for preventing bacteria from spreading throughout the body in sepsis. However, when a severe infection triggers a systemic hyperinflammation, it leads to diffuse microvascular thrombus, resulting in DIC (Dernaika and Kinasewitz, 2008). In the present study, platelet counts were significantly reduced by LPS. NVP-AUY922 pretreatment failed to improve the reduction in platelet count (Figure 2A), and NVP-AUY922 was therefore not further evaluated to improve LPS-induced activation of the coagulation system. MIP-2 is the main chemotactic factor released by macrophages in LPS infection (Qin et al., 2017). The results of this experiment showed that the plasma concentrations of IL-1β, TNF-α, IL-6, and MIP-2 in the LPS group increased 6 h after LPS infusion, and the plasma concentrations of IL-1β and TNF-α in the NVP + LPS group were significantly lower than those in the LPS group (Figures 3A,B). Nevertheless, NVP-AUY922 pretreatment did not reduce the production of IL-6 and MIP-2 induced by LPS (Figures 3C,D). The incapability of NVP-AUY922 pretreatment in correcting IL-6 and MIP-2 induction requires further investigation. Previous studies have indicated a correlation between IL-6 and the production of fibrinogen (Kushner and Rzewnicki, 1994; Borrelli et al., 1996). While NVP-AUY922 was not able to block the production of IL-6 and therefore could not improve the coagulopathy caused by LPS in the present study.

When liver damage occurs, ALT is released into the blood, causing an increase in blood ALT (Zhong et al., 2016). The present study showed a significant increase in the plasma ALT concentration up to 6 h after LPS infusion. Pretreatment of NVP-AUY922 could significantly decrease liver damage by LPS (Figure 2B). In previous clinical trials, treatment with NVP-AUY922 alone or in combination with other chemotherapy drugs could be safe and efficacious in treatment of advanced liver cancer (Wang et al., 2016b). NVP-AUY922 may ameliorate endotoxin-induced liver damage by inhibiting HSP90 and inducing HSP70. When the glomerular filtration rate drops, less CRE is excreted into the urine, resulting in its accumulation in the blood, so the concentration of CRE is as an indicator of renal function (Mehta et al., 2007). The present study showed a significant increase in plasma CRE concentration in rats after LPS infusion, indicating the kidney injury. The plasma CRE in the NVP + LPS group was significantly lower than that in the LPS group (Figure 2C), demonstrating that NVP-AUY922 could reduce the kidney injury caused by LPS. It has been shown that the lack of HSP70 causes more severe renal tubular damage and increases mortality in an animal model of acute kidney injury by ischemia–reperfusion in HSP70 knockout mice (Wang et al., 2011). NVP-AUY922 inhibits HSP90 and subsequently inducing HSP70, which could ameliorate the kidney injury by endotoxemia. When myofibrillar membrane and muscle fibers are damaged in sepsis, the components of necrotic muscle such as LDH and CPK are released into the blood circulation system, leading to renal tubules obstruction and acute kidney injury (Zimmerman and Shen, 2013). The present study showed that the plasma concentration of CPK increased significantly 6 h after LPS infusion, but NVP-AUY922 pretreatment failed to reduce the damage to skeletal muscle caused by the inflammatory response to LPS (Figure 2D); however, the underlying mechanism requires further investigation. The concentration of plasma LDH increased significantly 6 h after LPS infusion, demonstrating tissue damage by sepsis. NVP-AUY922 significantly lower the plasma LDH as a marker of cell damage (Figure 2E). Previous study had suggested that the administration of the HSP90 inhibitor 17-DMAG induces HSP70 production and is effective at suppressing plasma LDH concentrations in endotoxemic rats (Wang et al., 2016a). Taken together, the protective role of NVP-AUY922 inhibits HSP90 in cell damage could be dependent on inducing HSP70.

Heat shock proteins have important interactions with inflammatory responses in infection. The activated NFκB enters the nucleus and binds to genes to promote transcription, which in turn generates pro-inflammatory cytokines such as TNF-α and IL-1β, resulting in an inflammatory response (Salminen et al., 2008). It has been suggested that heat shock proteins, as chaperone proteins, help immune cells to fight against external infectious threats. Thus, when the inflammatory response expands, the expression of HSP70 also increases to suppress the inflammatory response and prevent inflammation from causing damage to the body (Kim et al., 2018). It has also been indicated that heat shock proteins inhibit NFκB signaling in a time- and HSP70-dependent manner (Schell et al., 2005). Therefore, in the present study, a 20-h pre-administration was used to ensure that the time was sufficient to produce a large amount of HSP70.

In human acute myeloid leukemia (AML) cells, NVP-AUY922, as a multi-client chaperone molecule, inhibits the inflammatory response by suppressing AKT and NF-κB. HSP90 inhibitor exerts preferential cytotoxicity in tumor cells compare to normal cells. This preference makes HSP a potential therapeutic target in treatment of tumor (Walsby et al., 2013). In a study of isolated white blood cells from human blood, HSP70 overexpression was found to reduce the production of pro-inflammatory factors induced by LPS (Ferat-Osorio et al., 2014). In a study on mouse macrophages (RAW264.7), overexpression of HSP70 inhibited the NFκB signaling pathway and reduced the expression of iNOS and MIP-2 (Chen et al., 2006). In the present study, NVP-AUY922 pretreatment attenuated the inflammatory response caused by endotoxemia, inhibited the activation of NF-κB p65 and IκB in the heart, lung, and liver (Figures 4A,B), and reduced the expression of iNOS (Figure 4C). In organs, NVP-AUY922 induced HSP70 when administered alone (NVP group). In the NVP+LPS group, HSP70 expression in the heart, lung, and liver was significantly higher than that in the LPS group 6 h after LPS infusion (Figure 5A), and HSF-1 protein expression in the myocardial nucleus was higher in the NVP + LPS group than that in the LPS group (Figure 5B), but no statistical difference was reached. It could be speculated that NVP-AUY922 may increase the expression of downstream HSP70 protein through the increased expression of HSF-1. Taken together, NVP-AUY922 exerts anti-inflammatory effects by inhibiting HSP90 and inducing HSP70.

The generation of ROS and adhesion molecules increase in infection, and in addition to the increase in HSP70, which scavenges ROS and MMPs to produce antioxidant effects (Antonova et al., 2012); the antioxidant stress protein HO-1 is expressed in response to stress. HO-1 is an antioxidant enzyme that produces carbon monoxide (CO) as an inhibitor of NFκB when the heme is degraded by HO-1. HO-1 also inhibits pro-inflammatory cytokines such as IL-6 and TNF-α, and activates anti-inflammatory cytokines such as IL-10, thus leading to homeostasis in the inflammatory process (Ahmed et al., 2017). The present study showed that the expression of HO-1 in the heart, lung, and liver of the LPS group was significantly increased 6 h after LPS infusion, indicating the increased expression of HO-1 against antioxidant stress due to the injury caused by LPS in rats (Figure 5C). The NVP + LPS group showed significantly higher HO-1 expression in the lung than the LPS group, and there was no significant difference in HO-1 expression in the heart and liver compared with the LPS group (Figure 5C). A previous study in human monocytes has showed that overactivation of TLR4 by LPS and subsequent over-expression of TNF-α reduced HO-1 expression and exacerbated inflammation (Rushworth et al., 2005). NVP-AUY922 upregulates HO-1 production to exert its anti-inflammatory effects especially in the lungs.

The conversion of LC3-I to LC3-II has been used as an indicator of autophagy in previous studies (Ho et al., 2016). The present study showed that the NVP + LPS group exhibited significantly higher amounts of LC3-II than the LPS group in three organs (Figure 5D). This suggests that NVP-AUY922 pretreatment may prevent cell damage by activating cellular autophagy against LPS-induced inflammation. HSP70 could stabilize lysosomes and prevent the hydrolytic enzymes release and cell necrosis (Chatterjee and Burns, 2017). In a study of gastrointestinal stromal tumor cells, NVP-AUY922 inhibited the growth of tumor cells and induced autophagy (Hsueh et al., 2013). This suggests that NVP-AUY922 stabilizes lysosomes by inducing HSP70 and prevents lysosomes from releasing hydrolytic enzymes to cause cell necrosis.

There are several pathways which can trigger rat cardiomyocyte apoptosis and activation of caspase 3. The first is procaspase-9 activates Apaf-1/dATP/cytochrome c apoptotic bodies (Feng et al., 2018). The second is the NO/iNOS pathway (Chakravortty et al., 2001). Six hours after LPS infusion, the expression of caspase-3 significantly increased in the LPS group, indicating that LPS caused severe cellular damage triggering apoptosis. In contrast, in the NVP + LPS group, the expression of caspase-3 protein in the cardiomyocytes decreased significantly (Figure 5E). In addition, NVP decreased neutrophil infiltration in lung injury and degermation and necrosis of hepatocytes in liver injury induced by LPS (Figure 6). The results suggest that NVP protect cardiomyocytes from apoptosis and attenuated lung and liver injury induced by LPS.

The overexpression of HSP70 can inhibit apoptosis and increase tumor cell growth (Rérole et al., 2011). HSP70 inhibits BAX protein, preventing the release of proapoptotic factors from mitochondria (Stankiewicz et al., 2005). HSP70 also inhibits the binding of Apaf-1 and procaspase-9 to form apoptotic bodies (Saleh et al., 2000). HSP70 binds to death receptors DR4/5 on the cell membrane, preventing the Apo-2L/TRAL (TNF-related apoptosis-inducing ligand) from activating the death-inducing signaling complex and downstream caspase-3 (Guo et al., 2005). This suggests that NVP-AUY922 could inhibit multiple apoptotic pathways by increasing HSP70, thereby protecting cells from the inflammatory response by LPS.

Both NVP-AUY922 and 17-DMAG are HSP90 inhibitors that bind to the N-terminal ATP binding site of HSP90, inhibit the ATP hydrolase to prevent HSP90 activation. In contrast to 17-DMAG, NVP pretreatment did not reduce LPS-induced platelet loss and decrease the release of plasma IL-6 and CPK. In previous studies, pretreatment with quercetin, an HSP70 inhibitor, failed to improve the coagulation function, so 17-DMAG may improve coagulopathy through mechanisms other than HSP70 (Wang et al., 2016a). Therefore, in the present study, it was speculated that NVP-AUY922 might also not be able to improve the coagulopathy through HSP70. The reason why NVP could not inhibit IL-6 release remains to be investigated. On the other hand, 17-DMAG pretreatment significantly reduced CPK values 6 h after LPS administration, and in the previous literature, induction of HSP70 reduced rhabdomyolysis in an animal model of heat exhaustion (Phillips, 2003). The reason for the failure of NVP-AUY922 to exert protective effects in skeletal muscle in the present study is unclear. Whether it is due to insufficient expression of HSP70 in skeletal muscle remains to be further investigated. Both HSP90 inhibitors significantly reduced the inflammatory response in endotoxemic rats, but the antioxidant capacity of NVP-AUY922 was weak (based on its ability to induce HO-1), which may be one of the reasons why NVP-AUY922 is less effective than 17-DMAG at protecting skeletal muscle, increasing platelet counts, and reducing IL-6 in endotoxemia. It is worthy noticing that, in clinical trials, both NVP-AUY922 and 17-DMAG causes visual disturbance and retinal damage (Zhou et al., 2013).

Our findings have certain important implications for mechanistic information to the knowledge base regarding the protective effects of NVP-AUY922 on inflammatory events induced by LPS. However this is a very observational study and the cause relationship of NYP-AUY922 in the induction of HSP-70 requires further investigation. It would have been interesting to study the cause relationship by knocking down or pharmacological approaches.

**FIGURE 7:**
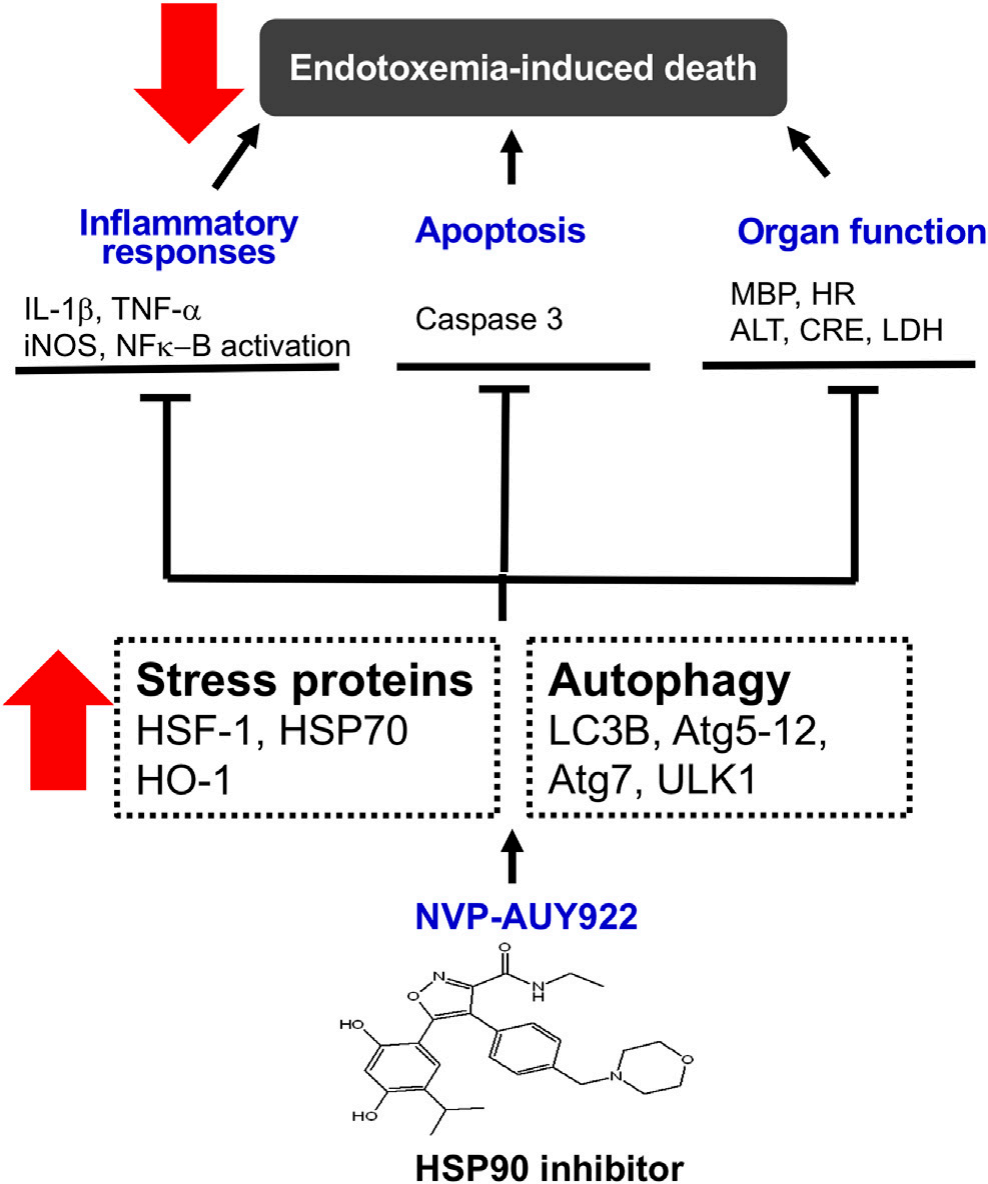
Schematic diagram of the present study. NVP-AUY922, a novel HSP90 inhibitor, is able to orchestrate and facilitate autophagy, which is HSP70 and HO-1 dependent. It inhibits inflammatory response, and apoptosis, attenuates organ dysfunction and subsequently decreases morality in endotoxemia.

In summary, NVP-AUY922, a next-generation HSP90 inhibitor, induces HSP70 and HO-1, and preserves cellular autophagy to ameliorate the inflammatory response, apoptosis, and multiple organ failure in endotoxemic rats (Figure 7).

## Data Availability

The raw data supporting the conclusions of this article will be made available by the authors, without undue reservation.
